# Draft genome sequence of *Trametes villosa* (Sw.) Kreisel CCMB561, a tropical white-rot Basidiomycota from the semiarid region of Brazil

**DOI:** 10.1016/j.dib.2018.04.074

**Published:** 2018-04-25

**Authors:** Dalila Souza Santos Ferreira, Rodrigo Bentes Kato, Fábio Malcher Miranda, Kenny da Costa Pinheiro, Paula Luize Camargos Fonseca, Luiz Marcelo Ribeiro Tomé, Aline Bruna Martins Vaz, Fernanda Badotti, Rommel Thiago Jucá Ramos, Bertram Brenig, Vasco Ariston de Carvalho Azevedo, Raquel Guimarães Benevides, Aristóteles Góes-Neto

**Affiliations:** a State University of Feira de Santana, Departament of Biological Science, Feira de Santana, BA 44036-900, Brazil; b Federal University of Minas Gerais, Institute of Biological Sciences, Belo Horizonte, MG 31270-901, Brazil; c Federal Center of Technological Education of Minas Gerais (CEFET-MG), Belo Horizonte, MG 30421-169, Brazil; d University of Göttingen, Institute of Veterinary Medicine, Burckhardtweg 2, D-37077 Göttingen, Germany; e Federal University of Pará, Institute of Biological Sciences, Belém, PA 66075-110, Brazil; f Federal University of Pará, Computer Science Graduate Program, Belém, PA 66075-110, Brazil

## Abstract

Herein, we present the draft genome of *Trametes villosa* isolate CCMB561, a wood-decaying Basidiomycota commonly found in tropical semiarid climate. The genome assembly was 57.98 Mb in size with an L50 of 691. A total of 16,711 putative protein-encoding genes was predicted, including 590 genes coding for carbohydrate-active enzymes (CAZy), directly involved in the decomposition of lignocellulosic materials. This is the first genome of this species of high interest in bioenergy research. The draft genome of *Trametes villosa* isolate CCMB561 will provide an important resource for future investigations in biofuel production, bioremediation and other green technologies.

## Specifications Table

**Table:** 

Subject area	Biology
More specific subject area	Mycology, Genomics, Biotechnology
Type of data	Genomic sequence, gene prediction and annotation of *Trametes villosa* isolate CCMB561
How data was acquired	The whole genome was sequenced with an Illumina Hi-Seq. 2500
Data format	Draft genome assembly and gene annotation
Experimental factors	The mycelium derived from field-collected basidiomata was cultured on potato dextrose agar (PDA) medium at room temperature and DNA was extracted with a FastDNA^TM^ Soil kit (MPBio)
Experimental features	The genome was assembled with SPAdes version 3.11.1 and annotated with MAKER version 2.31.9
Data source location	Basidiomata of *T. villosa* were collected in decaying wood (fallen branch) of an unidentified angiosperm in the Brazilian semiarid region (Serra das Candeias, Quijingue, Bahia, Brazil; Lat: 39°04'30"W and Long: 10°55'16''S).
Data accessibility	This Whole Genome Shotgun project has been deposited at DDBJ/ENA/GenBank under the accession number PUDQ00000000 (https://www.ncbi.nlm.nih.gov/nuccore/PUDQ00000000). The short reads have been deposited at SRA under the accession number SRR6763787_(https://www.ncbi.nlm.nih.gov/sra/?term=SRR6763787).

## Value of the data

• It is the first draft genome of *Trametes villosa*, a tropical white-rot Basidiomycota from the semiarid region of Brazil, promising for its production of ligninolytic enzymes.
• *T. villosa* isolate CCMB561 is a good producer of lignin peroxidase, manganese peroxidase and laccase, enzymes considered crucial for lignin degradation, providing a major advantage for its use in bioenergy research.
• The draft genome will accelerate functional genomics research, helping to understand the molecular basis of lignin decay by this fungus as well as advancing its enzymatic applications.

## 1 Data

The genus *Trametes* Fr. (Polyporaceae, Basidiomycota) comprises 20 species usually growing on decaying wood of angiosperms [1]. *Trametes* is morphologically characterized by its pileate basidiomata with a trimitic hyphal system and non-amyloid, non-dextrinoid and thin-walled spores, without hymenial cystidia [2].

*Trametes villosa* (Sw.) Kreisel is a common species in the Brazilian semiarid region [3]. It is a good producer of the three important ligninolytic enzymes: Laccase (Lac) [4], Manganese Peroxidase (MnP) [5] and Lignin Peroxidase (LiP) [6], demonstrating its high potential for biotechnological applications. However, little is known about the function and structure of *T. villosa* genes, which requires detailed investigation.

White-rot basidiomycotan fungi are the main producers of ligninases that substantially contribute to lignin decay of wood [7], [8]. Nowadays, ligninolytic enzymes of white-rot fungi have been broadly studied for their potential applications in a wide range of industrial bioprocesses such as decolorization of industrial dyes, the pulp bleaching of paper, textile industry and the degradation of organopollutants [9]. Furthermore, *T. villosa* simultaneously produces LiP, MnP and Lac [5], [6], [10] whereas other lignin decay fungi produce only one or two of these ligninolytic enzymes simultaneously [11], [12]. Thus, a species able to produce the three ligninolytic enzymes in the same bath culture is highly desirable for biotechnological applications [6].

In order to accelerate the studies on functional genomics and elucidate molecular processes of lignin decay in this species, the genome of *T. villosa* CCMB561 was sequenced and assembled. Sequencing was performed using the paired-end method with the Illumina HiSeq. 2500, which generated 25,034,256 reads with a mean read length of 151 bp and a total of 7.5 Gbp of data. The resulting genome assembly of *T. villosa* CCMB561 contained 57.98 Mb, which was larger than the 33.6 Mb genome of *Trametes hirsuta* [13], which is the phylogenetically closest species with available complete genome based on a five-marker dataset [14]. According to QUAST version 4.4 [15], the assembled draft genome of *T. villosa* CCMB561 consisted of 10,323 contigs (6161 longer than 1 kbp), with N50 of 16.5 kb and L50 of 691, while the largest contig spanned 647,839 bp, and the GC content of the genome was predicted as 59.34% (Table 1). A total of 16,711 genes were predicted, encoding proteins with an average length of 496 amino acids. The CAZymes analysis identified 590 these genes encoding carbohydrate-active enzymes (CAZymes), which included 237 glycoside hydrolases (GHs), 78 glycosyltransferases (GTs), 12 polysaccharide lyases (PLs), 69 carbohydrate esterases (CEs) and 112 auxiliary activities (AAs). Additionally, the genome of *T. villosa* CCMB561 was predicted to contain 820 proteins with oxidoreductase activity and 45 with peroxidase activity.

**Table 1:** Comparison of the genomic features of *Trametes villosa* isolate CCMB561 with *Trametes hirsuta* strain 072 [13].

**Organism**	**DB accession number**	**Isolation source**	**Contigs/Scaffolds**	**Genome size (Mb)**	**G + C (%)**	**CDSs/ORFs**
*Trametes villosa* CCMB561	PUDQ00000000	Decaying Wood	10,323	57.98	59.34	16,711
*Trametes hirsuta* 072	LIYB00000000	Soil	141	33.62	57.6	14,598

Although *T. villosa* CCMB561 commonly exists as an efficient wood decomposer, with a lignocellulolytic enzyme system mainly comprising laccases, lignin peroxidases and Mn-dependent peroxidases as well as a series of CAZymes [5], [6], [16], there are limited data about their synthesis, genetic coding and regulation. In the context of basic research, the genome sequencing of *T. villosa* CCMB561 presented herein will enrich the portfolio of potential genes, enzymes and pathways involved in the lignin degradation processes of the white-rot fungi. Additionally, in an applied context, the draft genome assembly of *T. villosa* CCMB561 will facilitate the development of ligninolytic enzyme production for biotechnological applications. Altogether, these efforts will ultimately provide important management tools to be used in industry, especially for lignocellulosic waste management.

## 2 Experimental design, materials and methods

### 2.1 Genomic DNA extraction and sequencing

The mycelium of *T. villosa* was grown on PDA medium for 5–7 days, at room temperature and after covering the superficial area of a 9-mm diam. Petri dish, it was scrapped. Genomic DNA was extracted with a FastDNA^TM^ Soil kit (MPBio). The quality and quantity of the genomic DNA were assessed by agarose gel electrophoresis and fluorometric analysis, respectively. A 450 bp library was prepared from genomic DNA with the NEBNext Fast DNA Fragmentation and Library Preparation Kit (New England Biolabs, Ipswich, NE, USA) following the manufacturer's instructions. Library quality was evaluated with Agilent 2100 Bioanalyzer. Whole genome sequencing was performed using an Illumina HiSeq. 2500.

### 2.2 Genome assembly and annotation

**Fig. 1:**
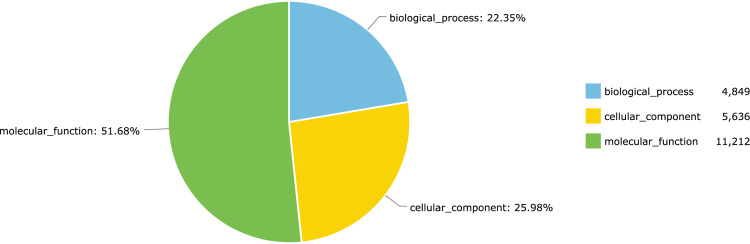
Groups of ontology.

Sequence read quality was assessed using FastQC v0.11.5 [17], while low quality bases were trimmed (Phred < 20) and overlapping sequences were collapsed with AdapterRemoval v2 [18]. We assembled the genome using SPAdes version 3.11.1 [19] with *k*-mers 49, 51, 53, 55, 57, 59, 61, 63, 65 and 67, which were estimated by KmerStream version 1.1 [20]. We identified 16,711 protein-coding genes that were predicted using MAKER2 version 2.31.9 [21], with support from *ab initio* predictors SNAP version 2006-07-28 [22] and gene prediction program. Fungal proteins, especially of the order Polyporales were also used to support gene prediction, by providing protein homology evidence. The contigs predicted by MAKER2 were analyzed with GoFeat [23] using the following databases: UNIPROT [24], INTERPRO [25], PFAM [26], SEED [27], NCBI [28], EMBL [29], KEGG [30]. CAZymes were identified with dbCAN version 5.0 [31]. The GoFeat analysis classified *T. villosa* 16,711 predicted genes in three groups of ontologies (biological process, cellular component and molecular function): 22.35% in biological process, 25.98% in cellular component and 51.68% in molecular function (Fig. 1). GO terms in biological process group are molecular events related to cell functioning, in cellular component are terms associated with their intra or extracellular location, and in molecular function group are elementary activities of the gene products at molecular level.

**Fig. 2:**
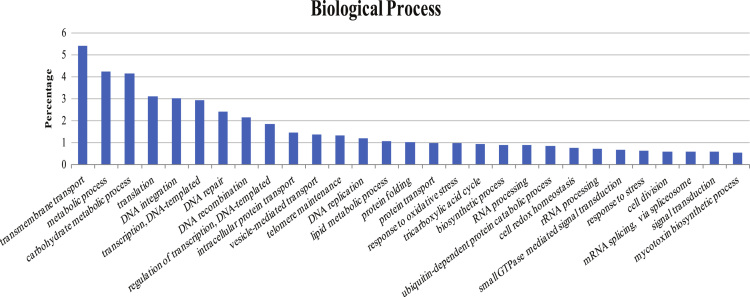
The histogram shows the percentage of GOs in biological process group.

**Fig. 3:**
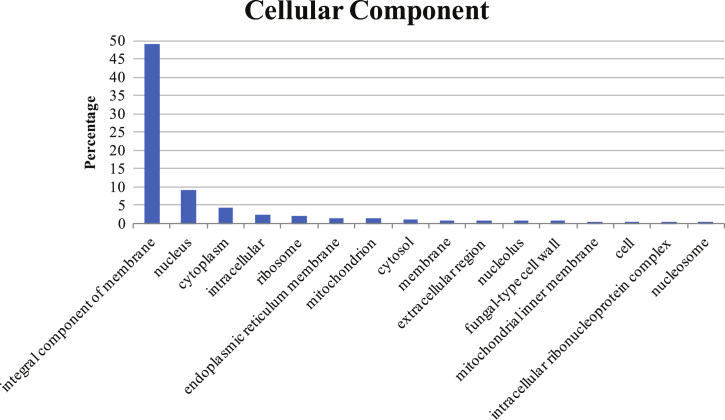
The histogram exhibits the percentage of GOs in cellular component group.

**Fig. 4:**
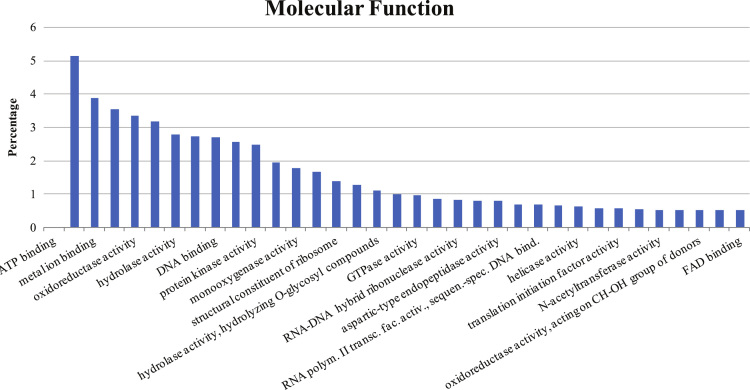
The histogram depicts the percentage of GOs in molecular function group.

For more details, we selected the most frequent gene ontologies (GOs) terms in each group to be represented in Fig. 2, Fig. 3, Fig. 4.
