# Supplementary material for: Draft genome sequence of Trametes villosa (Sw.) Kreisel CCMB561, a tropical white-rot Basidiomycota from the semiarid region of Brazil
Source: Data Brief. 2018 Apr 25;18:1581–7. doi: 10.1016/j.dib.2018.04.074 (PMC5998210; doi:10.1016/j.dib.2018.04.074)
Supplement: Supplementary file 1 — Supplementary material [file mmc1.docx]

**Conflict of Interest Statement**

The authors declare that the research was conducted in the absence of any commercial or financial relationships that could be construed as a potential conflict of interest.
